# Mitochondrial Calcium Uptake Modulates Synaptic Vesicle Endocytosis in Central Nerve Terminals*

**DOI:** 10.1074/jbc.M115.686956

**Published:** 2015-12-07

**Authors:** Jamie Roslin Keynes Marland, Philip Hasel, Katherine Bonnycastle, Michael Alan Cousin

**Affiliations:** From the Centre for Integrative Physiology, George Square, University of Edinburgh, Edinburgh EH8 9XD, Scotland, United Kingdom

**Keywords:** calcium, endocytosis, mitochondria, neuron, synapse, vesicles

## Abstract

Presynaptic calcium influx triggers synaptic vesicle (SV) exocytosis and modulates subsequent SV endocytosis. A number of calcium clearance mechanisms are present in central nerve terminals that regulate intracellular free calcium levels both during and after stimulation. During action potential stimulation, mitochondria rapidly accumulate presynaptic calcium via the mitochondrial calcium uniporter (MCU). The role of mitochondrial calcium uptake in modulating SV recycling has been debated extensively, but a definitive conclusion has not been achieved. To directly address this question, we manipulated the expression of the MCU channel subunit in primary cultures of neurons expressing a genetically encoded reporter of SV turnover. Knockdown of MCU resulted in ablation of activity-dependent mitochondrial calcium uptake but had no effect on the rate or extent of SV exocytosis. In contrast, the rate of SV endocytosis was increased in the absence of mitochondrial calcium uptake and slowed when MCU was overexpressed. MCU knockdown did not perturb activity-dependent increases in presynaptic free calcium, suggesting that SV endocytosis may be controlled by calcium accumulation and efflux from mitochondria in their immediate vicinity.

## Introduction

Action potential stimulation causes influx of extracellular calcium into central nerve terminals through voltage-gated calcium channels. This increase in [Ca^2+^]*_i_* triggers synaptic vesicle (SV)^2^ exocytosis (1) and modulates the kinetics of SV endocytosis (2). Alterations in the rate of calcium clearance from the nerve terminal both during and after stimulation can result in short-term plastic changes in the efficiency of neurotransmitter release (3) resulting from modified SV exocytosis and/or endocytosis kinetics. Calcium clearance occurs via several routes, including diffusion, extracellular extrusion by the plasma membrane Ca^2+^ ATPase and Na^+^/Ca^2+^ exchangers, and accumulation into mitochondria (4).

Mitochondrial calcium uptake can modulate neurotransmitter release during intense stimulation in both large atypical central nerve terminals (5) and neuromuscular junctions (6–8). At typical small central nerve terminals, evidence of an essential role for mitochondrial calcium uptake in the modulation of SV recycling is still absent. Comparisons between nerve terminals with or without mitochondria suggest no difference in the extent and kinetics of SV fusion (9–11). Furthermore, pharmacological inhibition of mitochondrial calcium uptake has no effect on SV fusion in isolated central nerve terminals (12). Therefore, it is unclear whether mitochondrial calcium uptake plays a direct role in modulating SV recycling during physiological stimulation.

Mitochondria accumulate [Ca^2+^]*_i_* into their matrix via the mitochondrial calcium uniporter (MCU), with uptake driven by the inner mitochondrial membrane potential (ΔΨ) (13). Until recently, mitochondrial calcium uptake could only be accessed experimentally by either pharmacological blockers of the MCU or depolarization of ΔΨ, both of which have off-target effects (14). Identification of the gene encoding the MCU channel subunit has allowed direct genetic intervention to determine its role (15, 16). In this study, we manipulated MCU expression in primary neuronal culture to determine whether mitochondrial calcium uptake directly affected SV recycling during physiological stimulation trains. We found that ablating mitochondrial calcium uptake accelerated SV endocytosis with minimal effect on activity-dependent presynaptic [Ca^2+^]*_i_* levels.

## Experimental Procedures

### 

#### 

##### Materials

Cell culture media and supplements were obtained from Life Technologies. Papain was from Worthington Biochemical. DL-2-amino-5-phosphonopentanoic acid sodium salt and 6-cyano-7-nitroquinoxaline-2,3-dione disodium salt were from Abcam. Bafilomycin A1 was from Cayman Chemical. All other reagents were from Sigma-Aldrich. Synaptophysin-pHluorin (syp-pHluorin) was from Prof. L. Lagnado (University of Sussex, UK). Mito-GCaMP2 was from Prof. X. Wang (Yunnan Center for Disease Prevention and Control, China). GCaMP6f (plasmid 40755) and SypHer-mt (plasmid 48251, referred to in this study as mito-pH) were from Addgene. Synaptophysin-mCerulean has been described previously (17). MCU knockdown and overexpression plasmids were from Prof. H. Bading (Interdisciplinary Centre for Neurosciences, Germany). The knockdown plasmids were based on an recombinant adeno-associated virus backbone and contained a calcium/calmodulin-dependent protein kinase II promoter driving mCherry expression and a U6 promoter driving shRNA expression (18). The knockdown plasmids targeted sequences within the 3′ UTR of the *Mcu* transcript (shMcu1, TAGGGAATAAAGGGATCTTAA; shMcu2, GGGCTTAGCGAGTCTTGTC; scrambled control, GTGCCAAGACGGGTAGTCA). The MCU plasmid expressed MCU fused to tDimer (18), whereas mCherry was expressed from pmCherryC1 (Clontech).

##### Cell Culture and Transfection

Primary cultures of dissociated hippocampal neurons were generated from C57Bl/6J mouse embryos of both sexes at embryonic day 17.5 (17) at a density of 5 × 10^4^ cells/coverslip. After 10–11 days *in vitro*, cultures were transfected with plasmids using Lipofectamine 2000 (Life Technologies) according to the instructions of the manufacturer and used 2 days after transfection for overexpression and 3 days after transfection for shRNA.

##### MCU Knockdown and Western Blotting

Primary cortical cultures were infected with an adeno-associated virus containing either shScr or shMcu1 at ∼10^11^ particles/μl. Cells were infected overnight after 4 days *in vitro*, and expression was verified by mCherry fluorescence at day *in vitro* 10. Infection efficiency was estimated to be between 80–90%. SDS-PAGE and Western blotting were performed using the Xcell Surelock system (Invitrogen) and precast gradient gels (4–20%) as described previously (18). Antibody concentrations were as follows: β-actin (1:2000, Abcam, catalog no. ab8227) and Mcu/ccdc109a (1:500, Sigma, catalog no. HPA016480). Secondary horseradish peroxidase-linked antibodies were used to visualize bands on Kodak X-Omat films. Blots were scanned digitally, and densitometric analysis was performed using ImageJ. Differences in protein loading were corrected using β-actin as a loading control on the same membrane.

##### Fluorescence Imaging

Coverslips were mounted in a Warner RC-21BRFS chamber containing embedded parallel platinum wires and bathed in imaging buffer containing the following: 119 mm NaCl, 2.5 mm KCl, 2 mm CaCl_2_, 2 mm MgCl_2_, 25 mm HEPES, 30 mm glucose, 0.01 mm 6-cyano-7-nitroquinoxaline-2,3-dione disodium salt, and 0.05 mm DL-2-amino-5-phosphonopentanoic acid sodium salt adjusted to (pH 7.4). For experiments examining exocytosis kinetics and SV pool size, the imaging buffer also contained 1 μm bafilomycin A1 to inhibit the acidification of retrieved SVs. For experiments examining SV acidification kinetics, cultures were exposed to acidic imaging buffer (substituting 25 mm MES for HEPES (pH 5.5)) for defined intervals. Cultures were stimulated electrically with a train of action potentials delivered at 10 Hz (1-ms pulse width, 100-mA constant current output). Experiments were performed at room temperature unless indicated otherwise (for experiments at 37 °C, a constant temperature was maintained using an in-line heating system (Warner Instruments)).

Imaging was performed on a Zeiss Axio Observer D1 epifluorescence microscope using a ×40, 1.3 numerical aperture oil immersion objective. Wavelength settings were as follows: mito-pH, GCaMP6f, and syp-pHluorin, 480-nm excitation, 530/40-nm bandpass emission; mCherry, either 550-nm excitation, 630/60-nm bandpass emission or 556/25-nm excitation, 630/98-nm bandpass emission; synaptophysin-mCerulean, 430-nm excitation, 530/40-nm bandpass emission; and mito-GCaMP2, 470/27-nm excitation, 512/30-nm bandpass emission. All images were acquired at 4-s intervals on a Hamamatsu Orca-ER camera, except for mito-GCaMP2, where a Zeiss AxioCam 506 mono camera with 2 × 2 pixel binning was used.

##### Data Analysis and Statistics

Image processing and analysis was performed using FIJI. Time series images of transfected neurons were aligned using the StackReg plugin (19). Regions of interest were placed over presynaptic terminals (syp-pHluorin and GCaMP6f) that responded to stimulation or axonal mitochondria (mito-GCaMP2 and mito-pH). Fluorescence intensity in each region of interest was measured using the Time Series Analyzer plugin (http://rsb.info.nih.gov/ij/plugins/time-series.html). Data from regions of interest on each coverslip were then normalized to resting baseline fluorescence and averaged to give mean F/F_0_ fluorescence. For syp-pHluorin data, this was normalized further to either the peak fluorescence during stimulation or following application of alkaline buffer (50 mm NH_4_Cl exchanged for 50 mm NaCl). Non-linear regression was used to fit a monoexponential function to the post-stimulation syp-pHluorin fluorescence data to derive the time constant (τ) of endocytosis or to the syp-pHlourin response in acidic imaging buffer to derive the τ of SV acidification. For SV endocytosis, exponentials were fitted between 4–108 s after termination of stimulation. This monoexponential decay fit all of our data extremely well (R^2^ = 0.986, *n* = 95 separate experiments, S.D. = 0.02). The τ for SV acidification was obtained by fitting a monoexponential decay to syp-pHluorin traces during quenching with MES buffer after stimulation. This allowed for an accurate determination of τ even at relatively slow acquisition rates. One-way and two-way ANOVA were used to compare differences between either groups or time series data (using Holm-Šídák post hoc tests). Sample size (*n*) was the number of independent coverslips analyzed. All data and statistical analyses were performed using Microsoft Excel and GraphPad Prism 6.

## Results

**FIGURE 1.**
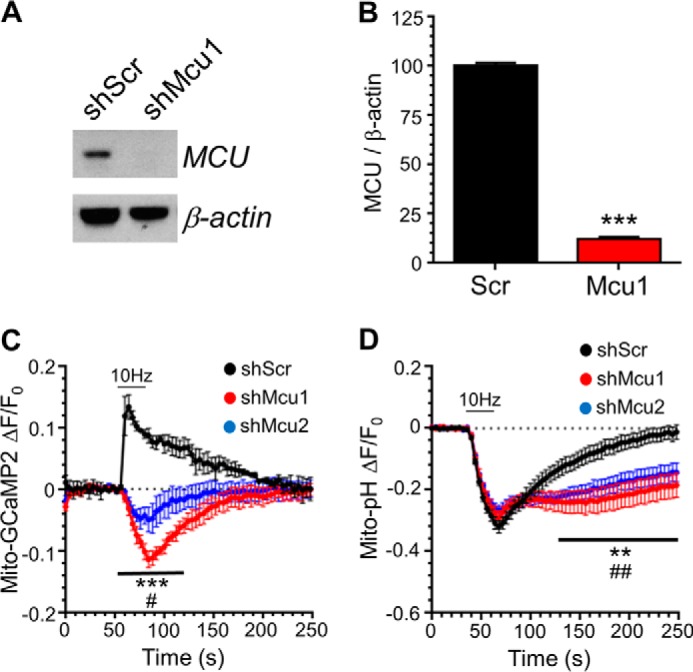
**MCU knockdown ablates axonal mitochondrial Ca^2+^ uptake during neuronal activity.** *A* and *B*, neurons were infected with adeno-associated virus containing either shRNA targeting MCU (*shMcu1*) or a scrambled control (*shScr*) for 6 days. *A*, representative Western blots showing the relative levels of both MCU and β-actin (loading control). *B*, quantification of MCU knockdown expressed as a percentage of shScr (*MCU*/β*-actin*). *n* = 3 independent cultures; ***, *p* < 0.001; Students *t* test. *C* and *D*, neurons transfected with either mito-GCaMP2 or mito-pH and either shRNA targeting MCU or a scrambled control were stimulated with an action potential train (10 Hz 30 s, indicated by the *bar*). *C*, average traces for mito-GCaMP2 fluorescence under each condition normalized to baseline fluorescence (Δ*F*/*F_0_*) ± S.E. (shScr *n* = 3, shMcu1 *n* = 4, shMcu2 *n* = 5). *D*, average traces for mito-pH fluorescence in each condition normalized to baseline fluorescence ± S.E. (shScr, *n* = 7; shMcu1, *n* = 7; shMcu2, *n* = 8). *C* and *D*, two-way ANOVA with Holm-Šídák post hoc tests; shMcu1: ***, *p* < 0.001; **, *p* < 0.01; shMcu2: ##, *p* < 0.01, #, *p* < 0.05; both to Scr control.

Presynaptic mitochondria have been proposed to modulate SV recycling via the accumulation of calcium during intense stimulation conditions (5, 6, 8, 10). Because evidence for such a role is still lacking in typical small central nerve terminals during physiological stimulation, we employed a genetic strategy to manipulate mitochondrial calcium uptake in primary neuronal culture. MCU expression was ablated using independent shRNA constructs validated previously (18). Knockdown of MCU was highly efficient, with shRNA against MCU reducing its expression to 12% ± 1% compared with a scrambled control shRNA in cortical cultures (Fig. 1*A*). To also demonstrate a functional effect of MCU knockdown in our hippocampal culture system, in which all subsequent experiments were performed, the evoked increase in mitochondrial free calcium was monitored using the genetically encoded reporter mito-GCaMP2 (20). Neurons expressing scrambled shRNA displayed a rapid increase in fluorescence on challenge with an action potential train (10 Hz, 30 s) that gradually recovered to baseline after stimulation, indicating mitochondrial calcium uptake followed by efflux. MCU knockdown with two independent shRNAs ablated mitochondrial calcium uptake; the peak mito-GCaMP2 response was abolished in neurons expressing either shRNA (Fig. 1*C*). The mito-GCaMP2 reporter displayed a slow decrease in fluorescence during stimulation in MCU knockdown neurons, likely because of its quenching via a transient drop in mitochondrial matrix pH after activity-dependent cytosolic acidification (21–23). Consistent with this, neurons expressing the genetically encoded matrix pH reporter mito-pH (24) displayed a decrease in fluorescence during stimulation in both control and MCU knockdown neurons, which was very similar in kinetics to the mito-GCaMP2 response in MCU knockdown neurons (Fig. 1*D*). Therefore, MCU knockdown results in arrest of mitochondrial calcium accumulation in central nerve terminals.

**FIGURE 2.**
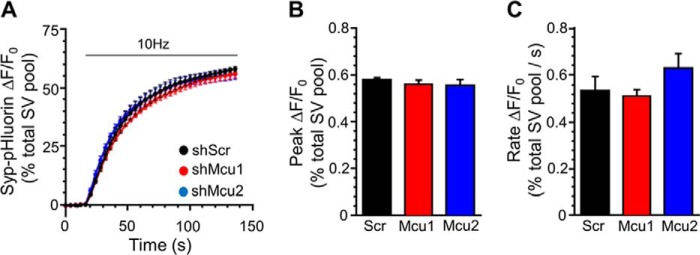
**MCU knockdown has no effect on SV exocytosis.** Neurons transfected with syp-pHluorin and either shRNA targeting MCU (*shMcu1* and *shMcu2*) or a scrambled control (*shScr*) were stimulated with an action potential train (10 Hz, 120 s, indicated by the *bar*) in the presence of bafilomycin A1. *A*, average traces for syp-pHluorin fluorescence (ΔF/F_0_ ± S.E.) under each condition normalized to maximal fluorescence obtained in alkaline buffer (total SV recycling pool). *B*, average peak Syp-pHluorin fluorescence normalized to the total SV recycling pool. C, mean SV exocytosis rate calculated using a linear fit to Syp-pHluorin fluorescence during the first 12 s of stimulation. *B* and *C*, data are mean ± S.E. (shScr, *n* = 6; shMcu1, *n* = 7; shMcu2, *n* = 5; one-way ANOVA with Holm-Šídák post hoc tests; all non-significant; *p* > 0.05).

To determine whether MCU knockdown affected SV recycling, we used the fluorescent reporter syp-pHluorin, which is a pH-sensitive variant of green fluorescent protein (pHluorin) fused within a luminal loop of the SV protein synaptophysin (25). The acidic SV interior quenches syp-pHluorin fluorescence. However, during SV exocytosis, its fluorescence is unquenched on exposure to the neutral pH of the extracellular medium. Syp-pHluorin also reports SV endocytosis as a fluorescence decrease because SV acidification occurs rapidly following SV retrieval (26). SV exocytosis can be isolated experimentally from endocytosis during stimulation by acute addition of the V-ATPase inhibitor bafilomycin A1, which prevents SV acidification (27). Stimulation of cultures expressing scrambled shRNA with an action potential train (10 Hz, 2 min) in the presence of bafilomycin A1 resulted in a rapid fluorescence increase, reflecting SV fusion (Fig. 2*A*). Knockdown of MCU with either shRNA did not affect the initial rate of SV fusion or the number of SV fusion events, monitored as a proportion of the total nerve terminal SV pool (revealed by neutralization of SV pH (Fig. 2, *B* and *C*)). Therefore, neither the rate nor the extent of SV exocytosis are affected by reduction of mitochondrial calcium accumulation.

**FIGURE 3.**
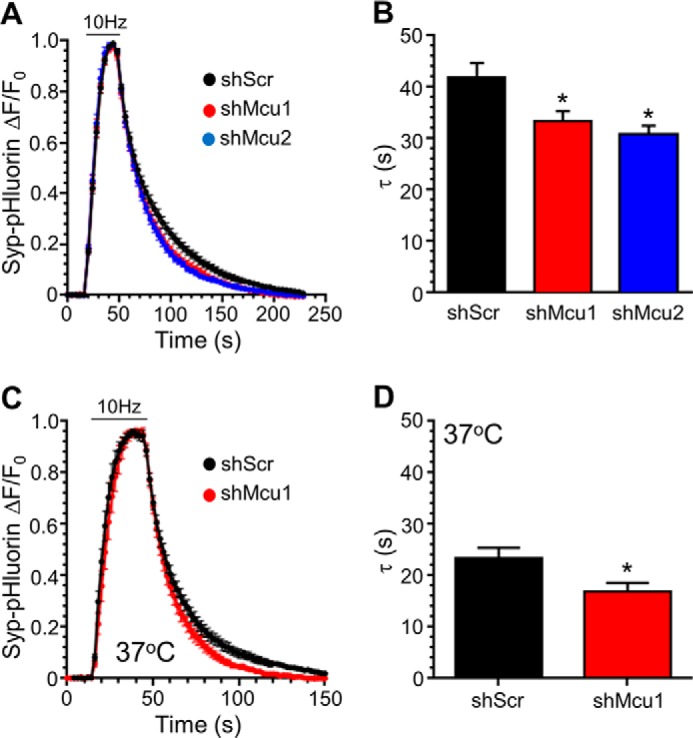
**MCU knockdown accelerates SV endocytosis.** Neurons transfected with syp-pHluorin and either shRNA targeting MCU (*shMcu1* and *shMcu2*) or a scrambled control (*shScr*) were stimulated with an action potential train (10 Hz, 30 s, indicated by the *bar*). *A*, average traces for syp-pHluorin fluorescence (ΔF/F_0_ ± S.E.) under each condition normalized to maximal fluorescence during stimulation (both shMcu traces are significantly different from scrambled shRNA over the range of 72–136 s; *p* < 0.05, two-way ANOVA). *B*, average syp-pHluorin τ ± S.E. (shScr, *n* = 14; shMcu1, *n* = 9; shMcu2, *n* = 7; one-way ANOVA with Holm-Šídák post hoc tests; *, *p* < 0.05). *C*, experiments were performed in an identical manner as in *A* apart from the temperature, which was set to 37 °C. *D*, average Syp-pHluorin τ ± S.E. (shScr, *n* = 10; shMcu1, *n* = 7; Student's *t* test; *, *p* < 0.05).

Increased [Ca^2+^]*_i_* modulates SV endocytosis in central nerve terminals (2). Therefore, we next investigated whether MCU knockdown affected this event. Cultures were again co-transfected with syp-pHluorin and either of the two MCU shRNA knockdown constructs or a scrambled shRNA control. Delivery of an action potential train (10 Hz, 30 s) evoked a peak of syp-pHluorin fluorescence followed by a post-stimulation recovery to baseline, the speed of which reflects the kinetics of SV endocytosis (Fig. 3*A*). MCU knockdown by either of the two shRNA sequences significantly accelerated this fluorescence recovery compared with neurons expressing scrambled shRNA, indicating that the SV endocytosis rate increases when mitochondrial calcium uptake is arrested (Fig. 3, *A* and *B*).

Recent studies have proposed that the mode of SV endocytosis in central nerve terminals can be dictated by temperature (28). Therefore, we next determined whether this MCU-dependent control of endocytosis was still apparent at physiological temperatures. In neurons expressing scrambled shRNA, there was a robust acceleration of SV endocytosis at 37 °C, in agreement with previous studies (29, 30) (Fig. 3, *C* and *D*). In neurons where MCU expression was ablated, there was a further acceleration compared with the scrambled control (Fig. 3, *C* and *D*). Therefore, inhibition of mitochondrial calcium uptake accelerates SV endocytosis across a wide temperature range.

**FIGURE 4.**
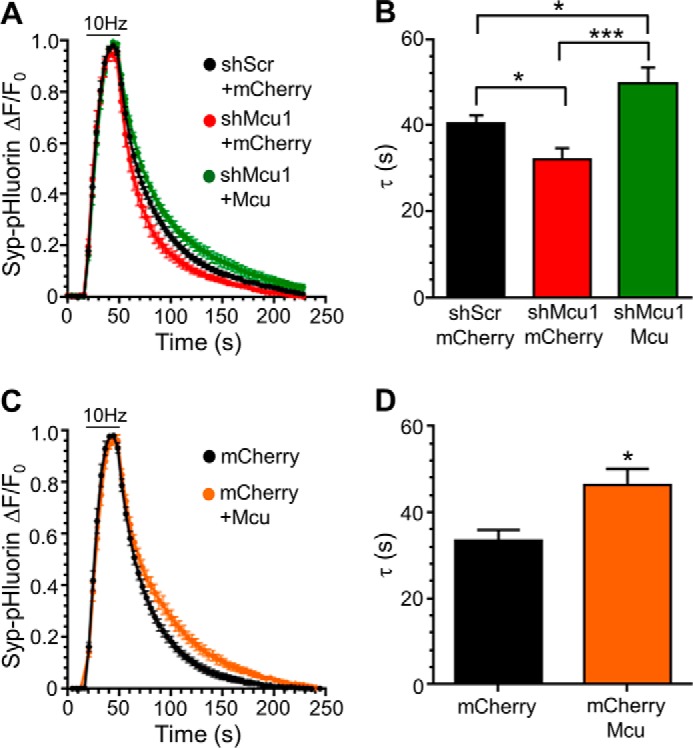
**MCU overexpression slows SV endocytosis.** *A* and *B*, neurons transfected with syp-pHluorin and either shRNA targeting MCU (*shMcu1*) or a scrambled control (*shScr*) and also MCU tagged with tDimer (MCU) or a control protein (mCherry) were stimulated with an action potential train (10 Hz, 30 s, indicated by the *bar*). *A*, average traces for syp-pHluorin fluorescence (ΔF/F_0_ ± S.E.) under each condition normalized to maximal fluorescence during stimulation. *B*, average syp-pHluorin τ ± S.E. (shScr + mCherry, *n* = 11; shMcu1 + mCherry, *n* = 9; shMcu1 + MCU, *n* = 8; one-way ANOVA with Holm-Šídák post hoc tests; ***, *p* < 0.001; *, *p* < 0.05). *C* and *D*, neurons transfected with syp-pHluorin and either MCU tagged with tDimer (MCU) or a control protein (mCherry) were stimulated with an action potential train (10 Hz, 30 s, indicated by the *bar*). *C*, average traces for syp-pHluorin fluorescence (ΔF/F_0_ ± S.E.) under each condition normalized to maximal fluorescence during stimulation. *D*, average syp-pHluorin τ ± S.E. (*n* = 9 for each; Student's *t* test; *, *p* < 0.05).

To confirm that the acceleration of SV endocytosis was not due to an off-target effect of the shRNA oligonucleotides, we performed rescue experiments with a shRNA-resistant form of MCU. Restoration of MCU expression fully reversed the acceleration of SV endocytosis observed in the presence of shRNA and significantly slowed kinetics compared with the scrambled shRNA control (Fig. 4, *A* and *B*). This result suggested that increasing the extent of calcium uptake into mitochondria via MCU overexpression may retard SV endocytosis. To test this, we determined the speed of SV endocytosis in neurons overexpressing MCU. Neurons with excess MCU displayed a significant slowing of SV endocytosis compared with expression of a control protein (mCherry) (Fig. 4, *C* and *D*). Therefore, modulation of mitochondrial calcium uptake has bidirectional effects on the speed of SV endocytosis.

**FIGURE 5.**
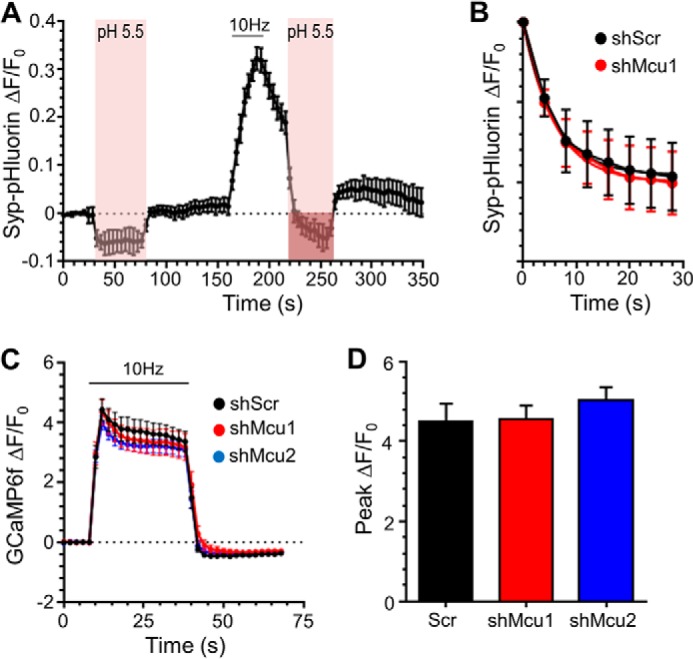
**MCU knockdown has no effect on SV acidification or evoked presynaptic [Ca^2+^]*_i_* increases.** *A* and *B*, neurons transfected with syp-pHluorin and either shRNA targeting MCU (*shMcu1*) or a scrambled control (*shScr*) were stimulated with an action potential train (10 Hz, 30 s, indicated by the *bar*). *A*, example trace displaying the syp-pHluorin response when cultures were exposed to a pulse of acidic buffer (pH 5.5) both before and after stimulation (indicated by the *pink region*). The *purple region* indicates the portion of the trace analyzed in *B. B*, average syp-pHluorin time course during post-stimulation acid exposure for either shScr or shMcu1 neurons ± S.E. (*n* = 4 for each). *C* and *D*, neurons transfected with GCaMP6f, synaptophysin-mCerulean, and shRNA targeting MCU or a scrambled control were stimulated with an action potential train (10 Hz, 30 s, indicated by the *bar*). *C*, average traces for presynaptic GCaMP6f fluorescence (ΔF/F_0_ ± S.E.) under each condition. *D*, average peak presynaptic GCaMP6f ΔF/F_0_ ± S.E. (*n* = 11 for all conditions, one-way ANOVA with Holm-Šídák post hoc tests, all non-significant, *p* > 0.05).

The apparent acceleration of SV endocytosis on ablation of mitochondrial calcium uptake may be due to increased SV acidification following cytosolic acidification, similar to that observed in the mitochondrial matrix (Fig. 1*D*). To determine this, we monitored SV acidification rates in neurons expressing scrambled or MCU shRNA by applying pulses of acidic buffer (Fig. 5*A*). This approach reveals this parameter by rapidly quenching the surface fraction of syp-pHluorin, allowing the τ of SV acidification to be visualized in recently retrieved SVs (26). SV acidification rates in neurons expressing scrambled shRNA were comparable with previous studies (26, 31), and knockdown of MCU had no significant effect on these kinetics (Fig. 5*B*; τ scrambled shRNA, 6.45 ± 1.10 s; shMcu, 6.60 ± 0.40; ns, *p* = 0.92; Student's *t* test; *n* = 4 for both conditions). Therefore, the enhanced fluorescence recovery rate reported by syp-pHluorin following MCU knockdown is a result of accelerated SV endocytosis kinetics.

Next we examined whether this acceleration was due to altered presynaptic [Ca^2+^]*_i_* levels during action potential stimulation. To determine this, we measured evoked [Ca^2+^]*_i_* increases using the fluorescent genetically encoded reporter GCaMP6f (32). GCaMP6f fluorescence was monitored in neurons expressing either shRNA against MCU or scrambled shRNA and also co-expressing synaptophysin tagged with mCerulean to unambiguously identify presynaptic sites. In neurons expressing scrambled shRNA, presynaptic GCaMP6f responded immediately to the action potential train (10 Hz, 30 s). This response plateaued for the duration of stimulation and then decreased rapidly following the end of stimulation as calcium was cleared from the presynaptic terminal (Fig. 5*C*). Knockdown of MCU with either shRNA had no effect on the peak presynaptic GCaMP6f response (Fig. 5*D*). This shows that knockdown of MCU expression causes no obvious difference in presynaptic calcium handling during action potential stimulation in small central nerve terminals.

## Discussion

Presynaptic calcium influx has key roles in triggering SV fusion and subsequent SV retrieval by endocytosis (1, 2). Furthermore, alterations in the rate of calcium clearance from the nerve terminal can evoke short-term plastic changes in the efficiency of neurotransmitter release (3). We present evidence that arrest of mitochondrial calcium accumulation accelerates SV endocytosis in small central nerve terminals.

We employed a knockdown approach to inhibit MCU activity in our culture system using two independent shRNA sequences that reduced mRNA and protein to background levels in primary neuronal cultures (18). Mitochondrial calcium uptake is completely dependent on the MCU because this event is ablated in mitochondria isolated from MCU knockout mice (33). We confirmed a similar functional effect of MCU knockdown by demonstrating abolition of mitochondrial calcium uptake using the reporter mito-GCaMP2 (20). During the course of these experiments, we also uncovered a transient activity-dependent acidification of the mitochondrial matrix because mito-GCaMP2 displayed a transient drop in fluorescence during stimulation in MCU knockdown neurons. This drop was most likely a consequence of activity-dependent cytosolic acidification (21–23) and the steep pH dependence of mito-GCaMP2 (20). This was confirmed by an independent reporter of matrix pH, mito-pH. Therefore, mitoGCaMP2 has limitations in accurately reporting matrix calcium dynamics. Regardless, a clear arrest of calcium accumulation can be observed during the initial stimulation period (first 10 s) in MCU knockdown neurons before changes in matrix pH occur.

Interestingly the recovery of matrix pH was retarded in neurons expressing MCU shRNA after stimulation terminated. One potential explanation for this is that arrest of mitochondrial calcium uptake reduces the rate of electron transport and, therefore, efflux of protons from the matrix. We suggest this is because mitochondrial calcium controls NADH production through several calcium-dependent enzymes in the tricarboxylic acid cycle and also pyruvate dehydrogenase dephosphorylation (34, 35). There is a possibility that this slower recovery in matrix pH may affect SV endocytosis by generating localized domains of low pH outside the mitochondrion. However, it should be noted that SV endocytosis is almost complete by the time the recovery of matrix pH is retarded in MCU knockdown neurons.

Previous work has employed pharmacological strategies to examine the role of mitochondrial calcium uptake in presynaptic function. However, these approaches have significant drawbacks. Depleting ΔΨ with mitochondrial uncouplers will inhibit ATP production, which is likely to have potent effects on presynaptic SV recycling (11, 22), and MCU blockers such as ruthenium red have off-target effects and limited cell permeability (14). Because our strategy involves the direct genetic modulation of MCU expression, this allowed us to interrogate the role of mitochondrial calcium uptake on SV recycling directly for the first time.

MCU knockdown did not alter the rate or extent of SV exocytosis. This agrees with previous work showing that either action potential-evoked release of the dye FM1–43 or synaptobrevin-pHluorin responses are unaffected by the presence or absence of mitochondria in the nerve terminals of cultured neurons (9, 10). Furthermore inhibition of mitochondrial calcium uptake using ruthenium red in hippocampal synaptosomes had no effect on FM1–43 release (12). However, during repetitive stimulation, mitochondria are required for neurotransmitter release by supporting SV mobilization from the reserve pool to the readily releasable pool (8, 10). Our study provides strong evidence that mitochondrial calcium uptake does not directly affect the coupling of activity-dependent calcium influx to SV fusion during a single stimulus train.

We found that MCU knockdown accelerates SV endocytosis, whereas overexpression of MCU slows kinetics. To our knowledge, this is the first demonstration of a direct interaction between mitochondrial calcium uptake and SV endocytosis. Previous studies at invertebrate neuromuscular junctions have suggested that SV endocytosis is unaffected by the absence of mitochondria (8). However, the approaches employed would not have captured the subtle modulation of speed observed in this study.

We found no alteration of evoked presynaptic [Ca^2+^]*_i_* increases upon knockdown of MCU. This was not due to saturation of GCaMP6f because stimulation in the presence of increased extracellular calcium or addition of a calcium ionophore increased the plateau response (data not shown). The lack of effect on evoked presynaptic [Ca^2+^]*_i_* increases conflicts with studies using tetanic stimulation at both neuromuscular junctions and large atypical central nerve terminals (5, 6, 8) but agrees with studies where nerve terminals are challenged with physiological trains of stimuli (10, 36). Our results suggest that mitochondrial calcium uptake does not play a major role in calcium clearance during physiological action potential stimulation at small central terminals.

How does MCU knockdown affect SV endocytosis, if not via control of bulk presynaptic [Ca^2+^]*_i_*? One potential explanation is that mitochondria may buffer [Ca^2+^]*_i_* in specific presynaptic regions close to sites of SV endocytosis, such the periactive zone. In support, mitochondria are typically found in close apposition to calcium channels so that the high local calcium concentrations at the mouth of the channel overcome the low affinity of the MCU (13). This mitochondrial calcium buffering should not affect SV fusion because the synchronous [Ca^2+^]*_i_* increase at the active zone will saturate the calcium fusion machinery within the immediate vicinity of calcium channels (1). However, the potentially close physical localization of mitochondria to SV endocytosis sites may allow modulation after action potential stimulation is complete. In this model, mitochondria sequester calcium during stimulation with a post-stimulation calcium efflux, retarding the speed of SV endocytosis. In support, action potential stimulation-evoked calcium influx is proposed to retard SV endocytosis after an initial brief acceleration period (37–39), and chelation of active zone calcium microdomains enhances SV endocytosis (40). An alternative possibility is that mitochondrial ATP production is altered by MCU knockdown because SV recycling is highly dependent on oxidative phosphorylation (22, 41). However, this is unlikely to explain our observed effects on SV endocytosis because mitochondrial calcium uptake stimulates ATP production in nerve terminals during action potential firing (42).

In summary, our results show that activity-dependent calcium uptake into mitochondria selectively modulates SV endocytosis kinetics in central nerve terminals, suggesting that the location of mitochondria within the presynapse is critical for their role in modulating SV endocytosis. These findings also have implications for a number of human pathological conditions where mitochondrial calcium uptake is affected (43, 44).

## Author Contributions

J. R. K. M. and M. A. C. designed the experiments and wrote the manuscript. J. R. K. M., P. H., and K. B. performed the experiments and analyzed the data.

## Footnotes

2 The abbreviations used are:

SV: synaptic vesicle
MCU: mitochondrial calcium uniporter
syp-pHluorin: synaptophysin-pHluorin
ANOVA: analysis/analyses of variance.
